# From Pre-Swelling to Performance Enhancement: Mechanisms and Effects of an Instant Ultra High-Performance Bituminous Material Modifier

**DOI:** 10.3390/ma19030633

**Published:** 2026-02-06

**Authors:** Yuanyuan Li, Haowen Ji, Chonghui Wang, Derun Zhang, Fu Wang, Gangping Jiang, Jiahui Deng, Junjie Ke

**Affiliations:** 1 School of Civil Engineering and Architecture, Wuhan Institute of Technology, Wuhan 430074, China; liyy@wit.edu.cn (Y.L.); jhw0616@outlook.com (H.J.); wangfu@wit.edu.cn (F.W.); 22504010131@stu.wit.edu.cn (J.D.); kjj2027203879@outlook.com (J.K.); 2 School of Engineering and Innovation, Aston University, Birmingham B4 7ET, UK; 3 School of Civil and Hydraulic Engineering, Huazhong University of Science and Technology, Wuhan 430074, China; derunzhang@hust.edu.cn; 4 School of Transportation, Changsha University of Science and Technology, Changsha 410114, China; 25001010006@csust.edu.cn

**Keywords:** instant modifiers, dry-process modification, high-performance modified bitumen, pre-swelling mechanisms, rapid swelling, composite polymer

## Abstract

**Highlights:**

**What are the main findings?**

- Pre-swelling via 120 min shearing with 3 h maturation forms a distinct bitumen-polymer spherical structure.
- SHVE-MB preparation is highly efficient, requiring only 10 min shearing without extra swelling or maturation.
- The bituminous material prepared by SHVE-M exhibits performance equivalent to that of wet-process.

**What are the implications of the main findings?**

- The proposed method significantly simplifies the production process of high-performance modified bitumen.
- It effectively bridges the performance gap between instant and traditional wet-process modified bitumen.
- The findings provide a more time-efficient and robust solution for road engineering materials.

**Abstract:**

To elucidate the modification and pre-swelling mechanisms of instant bituminous modifiers and their contribution to bituminous materials’ performance, this study investigates an instant ultra-high-performance bitumen modifier (SHVE-M). Fluorescence microscopy (FM), gel permeation chromatography (GPC), physical property tests, viscoelastic properties tests, dynamic shear rheometer (DSR), and mixture pavement performance tests were employed to systematically characterise the instant modified bitumen (SHVE-MB) and its mixture (SHVE-MBM). The results indicate that SHVE-M forms a stable “bitumen phase–polymer spherical phase” structure. ImageJ-win64 analysis revealed that SHVE-M exhibits a modifier area fraction of 46.68% and an average area fraction of 0.22‰, while SHVE-MB achieves a modifier area fraction of 17.54% and an average area fraction of 0.18‰. This morphology is supported by a large molecular size (LMS) content of 43% in SHVE-M. In terms of physical properties, the SHVE-MB (prepared via 10 min shearing) exhibited a penetration of 46.2 dmm, a softening point of 91.7 °C, and a ductility of 34.3 cm. These values are highly comparable to the conventional wet-process HVE-MB (prepared via 4 h maturation), with negligible differences of 0.5 dmm, 1.7 °C, and 1.4 cm, respectively. Quantitatively for viscoelasticity, SHVE-MB achieved a dynamic viscosity of 425,283.4 Pa·s at 60 °C and an elastic recovery rate of 92.1%, paralleling the 414,623.7 Pa·s and 93.6% of HVE-MB. Regarding mixture performance, the high-temperature dynamic stability (DS) of SHVE-MBM reached 7974 times/mm, approaching the 8256 times/mm of HVE-MBM. The water stability was excellent with a splitting tensile strength ratio (TSR) of 97.4% (vs. 98.0% for HVE-MBM). Furthermore, the low-temperature fracture toughness (K_IC_) reached 39.8 N/mm^1.5^, significantly outperforming SBS-MBM (27.9 N/mm^1.5^) and remaining close to HVE-MBM (43.9 N/mm^1.5^). These findings indicate that SHVE-MB effectively bridges the performance gap between instant and traditional high-viscosity modified bitumen, and the pre-swelling mechanism of SHVE-M is well characterized in this study.

## 1. Introduction

With the rapid expansion of the transportation sector, growing traffic volumes and increased axle loads from heavy vehicles have imposed more stringent demands on the performance of bituminous pavements [1]. To satisfy the modern requirements of road engineering, particularly in terms of temperature stability, fatigue resistance, and long-term durability, polymer-modified bitumen has emerged as one of the preferred solutions. Commonly used modifiers include styrene-butadiene-styrene (SBS) block copolymers, waste tire rubber powder, and resin-based polymers, etc. [2,3,4].

In the conventional wet-process modification (involving shearing and maturation), the interaction between polymer and bitumen is primarily achieved through mechanical shearing and extended high-temperature maturation [5]. As the most widely employed bituminous modifier, SBS triblock copolymer consists of polystyrene (PS) hard segments at both termini and a polybutadiene (PB) soft segment in the central region [6]. Based on molecular architecture, SBS is categorised into linear and star-shaped configurations. Linear SBS, characterised by a lower molecular weight, exhibits favourable solubility in bitumen, low viscosity, and enhanced processability and dispersion. In contrast, star-shaped SBS possesses a higher molecular weight, which confers greater modulus and improved high-temperature performance, albeit with more challenging dissolution in bitumen and stricter processing requirements [7,8]. These structural distinctions critically influence the rheological behaviour and storage stability of the resulting modified bitumen, which are key factors in the design and selection of polymer modifiers [9]. The performance enhancement of SBS-modified bitumen is primarily attributed to the polymer’s microscale multiphase structure and its ability to form physical crosslinks within the bitumen. Due to the thermodynamic incompatibility between the PS hard segments and the PB soft segments, SBS spontaneously undergoes microphase separation [10]. At ambient and elevated temperatures, the polystyrene segments aggregate into rigid, physically crosslinked domains dispersed within the polybutadiene matrix. These domains function as “anchors”, immobilising the elastomeric PB chains and thereby forming a three-dimensional network [11]. This structure provides mechanical reinforcement analogous to that of vulcanized rubber, while preserving the capacity for reversible melt flow upon heating [12]. As a result, this physical network endows SBS-modified bitumen with superior elastic recovery, enhanced fatigue resistance, and significantly improved resistance to high-temperature deformation [13]. During modification, SBS interacts with the base bitumen predominantly through swelling and the formation of a polymer network. Upon incorporation, SBS absorbs light fractions (particularly saturates and aromatics), leading to significant volumetric expansion. As the swelling process advances, the SBS polymer chains fully extend and become entangled, thereby establishing a continuous three-dimensional network within the bitumen [14]. This structural evolution alters the colloidal nature of the bitumen, effectively increasing the relative concentration of asphaltenes. Macroscopically, these changes manifest as reduced penetration, an elevated softening point, and a substantial increase in viscosity [15]. The network structure induced by swelling restricts the mobility of bituminous molecules and enhances cohesive strength, thereby significantly improving both high-temperature stability and low-temperature crack resistance, as well as elastic recovery properties [16].

The application of waste tire rubber powder as a modifier in road engineering provides dual advantages in environmental protection as well as resource recycling. It supports the current global trend towards green, low-carbon, and sustainable development, while simultaneously enhancing the critical performance of asphalt pavements, particularly high-temperature stability [17]. The chemical composition of rubber powder governs its intrinsic properties, which significantly influence the macroscopic behaviour of modified bitumen. Carbon black primarily affects the electrical conductivity and ageing resistance of the rubber powder [18]; meanwhile, rubber hydrocarbons facilitate vulcanisation, plasticisation, and anti-ageing processes during rubber production, and function as surfactants and softening agents within bituminous systems [19]. In its original status, natural rubber exhibits viscous fluid-like behaviour with high thermal sensitivity and limited elasticity, rendering it unsuitable for vehicle loads. Therefore, tires must undergo crosslinking via vulcanization to convert linear polymer chains into a three-dimensional network structure, thereby substantially improving mechanical strength as well as chemical stability [20]. When incorporated into bitumen, the components of rubber powder act synergistically: rubber hydrocarbon increases the softening point and enhances elastic recovery while reducing penetration; carbon black improves adhesion, durability, and resistance to abrasion; residual sulphur and anti-ageing agents contribute to enhanced high-temperature stability and oxidation resistance, respectively [21,22].

In the field of road engineering, resins are commonly classified into thermoplastic and thermosetting types according to their thermal behaviour [23]. The former—primarily polyolefin polymers with linear crystalline structures, which exhibit plasticity at elevated temperatures and rigidity at lower temperatures. These materials enhance the viscosity at ambient temperatures and improve the high-temperature stability of bituminous mixtures. Typical examples include low-density polyethylene (LDPE), ethylene–vinyl acetate copolymer (EVA), polypropylene (PP), and amorphous polyalphaolefin (APAO) [23,24,25]. In contrast, thermosetting resins such as phenolic, epoxy (EP), and unsaturated polyester (UP) resins develop high rigidity and excellent thermal stability upon curing, making them particularly suitable for enhancing pavement resistance to permanent deformation [26,27,28]. Research indicates that under combined thermal and mechanical shearing conditions, resin modifiers can achieve uniform dispersion within the bitumen in the form of particles or filaments, thereby forming partially physically or chemically crosslinked elastic networks [25,29]. This structure not only restricts the flow of bitumen but also absorbs wax and light fractions through swelling, thereby reducing wax content and significantly increasing bituminous viscosity while decreasing its temperature susceptibility. With respect to specific materials, PE effectively elevates the softening point and enhances rutting resistance, although it exhibits relatively poor low-temperature ductility. In contrast, EVA contains polar acetate groups that improve elasticity, flexibility, and compatibility with bitumen, thus enhancing both high-temperature stability and low-temperature performance while maintaining good workability [23,25]. Furthermore, EP, the most extensively studied thermosetting modifier, possesses a high molecular weight and excellent chemical stability. When combined with cost-effective curing agents such as aromatic amines, it can penetrate bituminous molecules, overcome micellar barriers, and form a stable three-dimensional interpenetrating network via chemical crosslinking [30].

Although conventional wet-process shear technology is mature, stable, and well-established, its dependence on specialised equipment, complex logistical requirements, and the risks of component segregation and performance degradation during thermal storage have emerged as critical bottlenecks hindering the industry’s transition toward green and low-carbon development [31]. To address the limitations in bitumen production efficiency and energy consumption, a procedure named dry-process modification has been introduced. This approach involves directly incorporating modifiers into the mixing plant, where the thermal and mechanical energy generated during aggregate blending rapidly disperses the modifier, thereby significantly streamlining the process and enabling an efficient “mix-and-use” operation [32]. The method leverages intense mechanical forces to pre-enhance the polymer’s rheological properties and dispersion capacity, allowing it to withstand the harsh and time-constrained mixing environment. Dry-process techniques can be classified into distinct pathways: instant modifiers are designed to quickly modify the bituminous binder to emulate the performance of conventionally modified bitumen, whereas broad-sense dry modifiers aim to improve both binder characteristics and overall bituminous mixture performance [33]. As a key subset of dry-process technology, instant modifiers pursue a qualitative advancement in binder performance under ultra-short processing durations.

Although instant technology delivers notable improvements in production efficiency, it confronts substantial challenges—both in practical engineering implementation and in underlying theoretical foundations. In practice, the macroscopic performance of instant-modified bitumen is frequently markedly inferior to that of conventional wet-process-modified bitumen [5]. This performance gap stems predominantly from the inability of current polymer formulations to achieve uniform and stable microstructures within the extremely limited processing time frame, thereby compromising key material properties. Theoretically, while the “shearing–swelling” mechanism governing conventional wet-process modification is well established, the microstructural phase evolution during instant technology—particularly at ultra-short time scales—remains poorly understood. To date, no systematic theoretical framework or mechanistic guidance has been developed to characterize or inform this process [34].

To address the aforementioned challenges, this study develops and focuses on an instant ultra-high-performance bituminous modifier (SHVE-M). In response to the current lack of fundamental theoretical guidance for dry-process modification, the pre-swelling mechanism of the instant modifier is systematically elucidated from the perspectives of micro-morphological phase evolution and molecular weight distribution, utilizing FM, Image J-based image processing, and GPC techniques. At the macroscopic level, the technical properties of the instant modified bitumen (SHVE-MB) are comprehensively evaluated through physical, viscoelastic, and rheological characterizations, while the pavement performance of the corresponding mixture (SHVE-MBM) is validated via rutting, moisture stability, and low-temperature semi-circular bending (SCB) tests. Crucially, a comparative analysis is conducted between the SHVE-M instant modification process and asphalt materials prepared using identical component ratios via the conventional wet-process, aiming to clarify the similarities and differences in modification efficacy between the two methods. This systematic approach, bridging micro-mechanisms and macro-responses, seeks to verify the feasibility of utilizing instant technology to achieve performance equivalent to or superior to conventional processes. The findings are intended to provide novel theoretical insights into the rapid dissolution and high-performance reconstruction of instant-modified bitumen, offering an advanced technical solution to the long-standing performance bottlenecks of dry-process modification.

## 2. Materials and Methods

### 2.1. Materials

#### 2.1.1. Raw Materials Composition of SHVE-M

**Figure 1:**
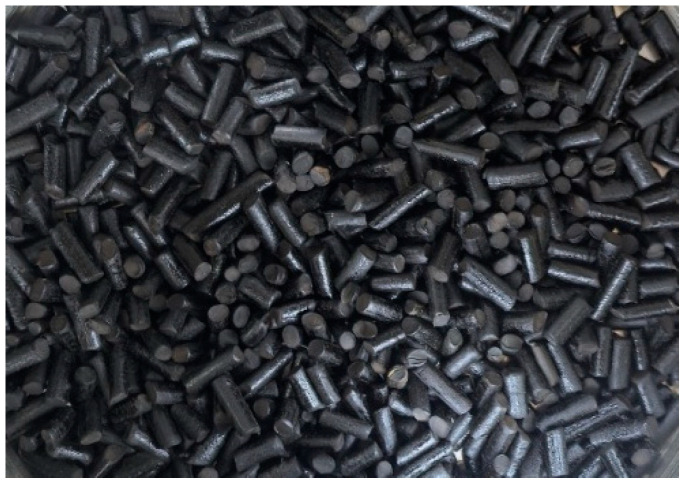
Apparent morphology image of SHVE-M.

SHVE-M, an instant ultra high-performance bitumen modifier, is made from 70# base bitumen, solubilizer, SBS, and a composite tackifier, as shown in Figure 1. Technical specifications of the raw materials are provided in Table 1, Table 2, Table 3 and Table 4. The composite tackifier is formulated from waste PE powder and stabiliser in a specific ratio.

#### 2.1.2. SBS-Modified Bitumen

The SBS-modified bitumen (SBS-MB) employed in this study was a commercially available product supplied by Hubei Guochuang Hi-tech Material Co., Ltd. in Wuhan, China. Containing approximately 4% SBS by mass. The relevant technical specifications are presented in Table 5.

#### 2.1.3. Aggregate

High-quality basalt was used as the aggregate. The properties of the coarse aggregate are presented in Table 6, and those of the fine aggregate are provided in Table 7.

#### 2.1.4. Filler

The filler serves the filling function within the bituminous mixture. The associated mineral powder was obtained from processed and finely ground limestone. The properties of mineral powder are presented in Table 8.

### 2.2. Preparation of SHVE-MB

**Figure 2:**
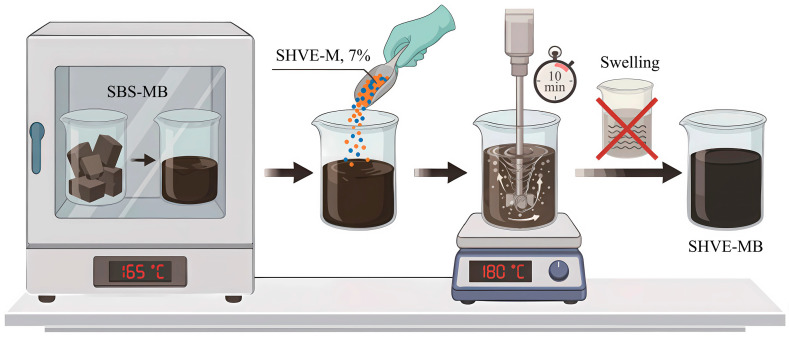
Preparation flow chart of SHVE-MB.

The preparation procedure for instant ultra high-performance modified bitumen (SHVE-MB) is presented in Figure 2, demonstrating a straightforward and practical methodology. As presented in Figure 2, the process consists of three steps: (1) Heat SBS-MB in a constant-temperature oven at 165 °C until it reaches a fully molten state; (2) Add SHVE-M to the SBS-MB at a dosage of 7% by mass of the SBS-MB; (3) Subject the mixture to mechanical shearing at 180 °C for 10 min using a high-speed shear device. Notably, no swelling or maturation stage is required, allowing SHVE-MB to be obtained directly.

### 2.3. Preparation of HVE-MB

**Figure 3:**
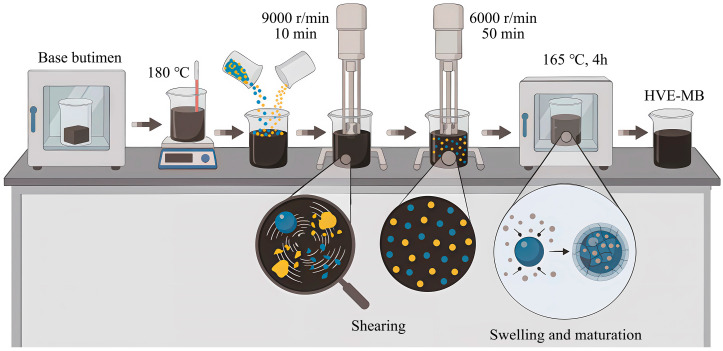
Preparation flow chart of HVE-MB.

High-viscosity and high-elasticity modified bitumen (HVE-MB) was prepared via the conventional shear maturation process for performance comparison with SHVE-MB, with the detailed procedure presented in Figure 3. As presented in Figure 3, the preparation consists of five sequential steps: (1) Heat the base bitumen in a constant-temperature oven at 135 °C until it is completely molten, then increase the temperature to 180 °C; (2) Gradually introduce accurately weighed modifiers in small, multiple batches to avoid localized agglomeration; (3) First, subject the mixture to continuous shearing at 9000 r/min for 10 min to achieve initial breakdown, wetting, and dispersion of the modifiers; (4) Subsequently, reduce the shear rate to 6000 r/min and continue shearing for an additional 50 min to further refine the modifier particles and ensure uniform distribution within the bitumen; (5) Finally, transfer the sheared mixture to a constant-temperature oven maintained at 165 °C for 4 h to facilitate complete absorption of light components (e.g., saturates and aromatics) by the modifiers, promoting full swelling and resulting in a stable HVE-MB.

The polymer modifier employed in HVE-MB is identical to that used in SHVE-MB (see Section 2.1.1), with each component incorporated at the same proportion as in SHVE-MB (e.g., SBS constitutes 6.3% of SHVE-MB; it is likewise blended at 6.3% in HVE-MB).

### 2.4. Preparation of Bitumen Mixture

#### 2.4.1. Gradation

**Figure 4:**
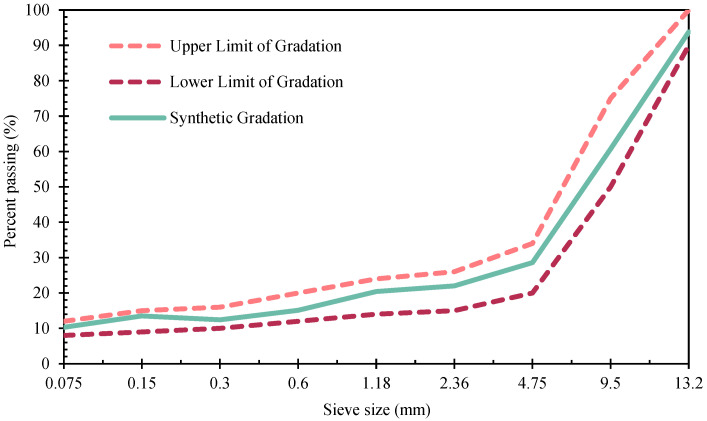
Gradation curve.

The aggregate gradation curve of the bituminous mixture employed in this study is presented in Figure 4. Following optimisation of the mix proportions using the Marshall method, the optimum bituminous content for both SHVE-MB and HVE-MB was determined to be 5.6%. To further improve structural stability and resistance to degradation, 0.4% lignin fibre (by total aggregate mass) was incorporated as a reinforcing agent during the mixing process.

#### 2.4.2. Mixing Process

The mixing procedures for the instant ultra high-performance modified bituminous mixture (SHVE-MBM), high-viscosity and high-elasticity modified bituminous mixture (HVE-MBM), and SBS-modified bituminous mixture (SBS-MBM) are as follows: (1) Preheat the aggregates to 190 °C and maintain this temperature for at least 6 h; simultaneously, heat the modified bitumen to 165 °C until it is completely molten; (2) Heat the mixing bowl to 190 °C, introduce the preheated aggregates, and mix for 90 s to ensure uniform distribution; (3) Add the lignin fibre and blend for 30 s. Subsequently, incorporate the modified bitumen and continue mixing for an additional 90 s to achieve homogeneous coating of the aggregates; (4) Add the mineral filler in the predetermined proportion and mix for 60 s.

### 2.5. Test Methods

#### 2.5.1. Fluorescence Microscope (FM)

FM employed in this study was the Model M330F-3M830F manufactured by Aoswei Co., Ltd. (Shanghai, China). A 420 nm blue-violet light source was used to irradiate the samples, exciting the hydrocarbons in the polymer to emit fluorescence, thereby generating images with a magnification of 100×.

ImageJ-win64 software was utilised to binarize the fluorescence images. To ensure the objectivity of the quantification and minimize the sensitivity of the results to manual thresholding, the “Auto Threshold” algorithm was employed for image segmentation. High-contrast representations were yielded in which the bitumen phase appeared in black and the polymer phase in white. For each sample, three parallel replicates were prepared to guarantee the statistical representativeness and reproducibility of the data. The modifier area fraction and the average particle area fraction were selected as key quantitative parameters. The former refers to the ratio of the total modifier area to the entire image area, while the latter is calculated by dividing the modifier area fraction by the number of microparticles, representing the mean area fraction per polymer microparticle.

#### 2.5.2. Gel Permeation Chromatography Test (GPC)

A Waters 1515 gel permeation chromatograph (Waters Corporation, Milford, MA, USA) was employed to determine the molecular weight and molecular weight distribution of polymers in SHVE-MB and HVE-MB samples. A Styragel HR series composite gel column was utilised, with chromatographic-grade tetrahydrofuran (THF)—a solvent with excellent solubility for both bitumen components (e.g., saturates, aromatics, asphaltenes) and polymers in SHVE-M (SBS, composite tackifier)—serving as the mobile phase. The flow rate was maintained at 1.0 mL/min, the column temperature was kept constant at 35 °C, and the injection volume was set to 20 μL. The detector temperature was maintained at the same level as the column temperature (35 °C), and each sample was eluted over a 30 min period.

#### 2.5.3. Physical Properties Tests

Softening point, penetration, and ductility tests were performed on SHVE-MB and HVE-MB samples. The softening point was determined using the ring-and-ball method in accordance with standard procedures. Two parallel samples were tested for each sample group, and the final result was the average value.

Ductility was evaluated at 5 °C with a constant stretching rate of 50 mm/min. Three parallel samples were tested for each sample group, and the final result was the average value.

Penetration was measured at 25 °C by allowing a standard 100 g needle to penetrate the bitumen vertically for a duration of 5 s, with the results reported in units of 0.10 mm. Each sample group was tested five times, with a test spacing of more than 1 cm, and the final result was the average value.

#### 2.5.4. Viscoelastic Properties Tests

A SYD-0620A dynamic viscosity tester (Shanghai Changji Geological Instrument Co., Ltd., Shanghai, China) was employed under a vacuum of 0.095 MPa and at a temperature of 60 °C. The viscosity was determined as the pcoroduct of the tube constant (calibration value ± 0.001) and the flow time (measured with an accuracy of 0.1 s). Two parallel samples were tested for each sample group, and the final result was the average value.

Elastic recovery tests were conducted on SHVE-MB and HVE-MB. Cylindrical specimens (5 cm in length and 1 cm in diameter) were stretched at a rate of 50 mm/min to a final length of 10 cm ± 0.25 cm and then halted. After being cut at the midpoint, the specimens were immersed in a water bath maintained at 25 °C for one hour. The residual length was subsequently measured, and the elastic recovery ratio was calculated using Formula (1):

(1) $$D=\frac{10-X}{10}\times 100$$

where D denotes the elastic recovery rate (unit: %), and X denotes the residual length (unit: cm).

#### 2.5.5. Dynamic Shear Rheology Test (DSR)

A Smart Pave 102 dynamic shear rheometer (Anton Paar GmbH, Graz, Austria) was employed to characterise the rheological properties of SHVE-MB and HVE-MB samples. Test conditions included operation in strain-controlled mode (with a strain accuracy of ±0.01%), a temperature sweep ranging from 52 °C to 100 °C (at a heating rate of 2 °C/min), a constant angular frequency of 10 rad/s, the use of a 25 mm parallel plate geometry, a sample thickness of 1.0 mm, and a strain amplitude of 0.5% to ensure measurements were conducted within the linear viscoelastic region.

#### 2.5.6. Rutting Test

A 300 mm × 300 mm × 50 mm rutting plate mold was employed to fabricate SHVE-MBM and HVE-MBM specimens through wheel-rolling compaction (SYD-0703-3, Shanghai Changji Geological Instrument Co., Ltd., Shanghai, China). After curing for two days under standard conditions (25 °C, 60% relative humidity), the specimens were transferred to a rutting tester (SYD-0719A, Shanghai Changji Geological Instrument Co., Ltd., Shanghai, China) and preheated at 60 °C for 5 h. The tests were then conducted continuously for 60 min under a vertical wheel load of 0.7 MPa and a rolling frequency of 42 cycles per min. The accumulated rut depth was recorded to determine the dynamic stability (DS value). Two parallel samples were tested for each sample group, and the final result was the average value.

#### 2.5.7. Water Stability Test

Standard Marshall specimens (101.6 mm in diameter and 63.5 mm in height), compacted with 75 blows on each side, were employed to evaluate the water stability of SHVE-MBM and HVE-MBM through immersion Marshall and freeze–thaw indirect tensile tests. Two parallel samples were tested for each sample group, and the final result was the average value.

Immersion Marshall test: Control specimens were immersed in a water bath maintained at 60 °C ± 0.5 °C for 30 min, whereas experimental specimens were subjected to immersion for 48 h under the same temperature conditions. Marshall stability was determined at a loading rate of 50 mm/min, and the residual stability ratio (MSR) was subsequently calculated.

Freeze–thaw splitting test: Experimental specimens were first vacuum-saturated at 0.09 MPa for 30 min, then frozen at −18 °C ± 1 °C for 16 h, followed by thawing in a water bath at 60 °C ± 0.5 °C for 24 h. Control specimens were maintained in a water bath at 25 °C ± 0.5 °C for 2 h. Indirect tensile strength was measured using a universal testing machine at a loading rate of 1 mm/min, and the freeze–thaw splitting tensile strength ratio (TSR) was calculated accordingly.

#### 2.5.8. Low-Temperature Semi-Circular Bending (SCB) Test

Standard Marshall specimens were split along their diameters to obtain SCB specimens. A pre-cut notch (5 mm in depth, 2 mm in width) was introduced at the centre of the bottom edge of each specimen. The specimens were conditioned in the constant-temperature chamber of the UTM-100 at −10 °C ± 0.5 °C for a duration of 4 h. A three-point bending test was performed using a 10 mm diameter loading platen at a displacement rate of 0.5 mm/min until specimen failure occurred. The test was terminated when the applied load decreased to below 0.1 kN. The maximum load attained during the test was recorded and used to calculate the fracture strength (Formula (2)) and fracture toughness (Formula (3)) [44].

(2) $$\sigma_{max}=\frac{F_{max}}{D\times T}$$

where $\sigma_{max}$ represents the fracture strength (unit: MPa); $F_{max}$ is the maximum load (unit: N); D is the specimen diameter (unit: mm); T is the specimen thickness (unit: mm).

(3) $$K_{IC}=\sigma_{max}Y_{1}\sqrt{\pi a}$$

where $K_{IC}$ represents the fracture toughness (unit: N/mm^1.5^); $Y_{1}$ is the stress intensity factor; a represents the notch length (unit: mm).

## 3. Results and Discussion

### 3.1. Pre-Swelling Mechanism

#### 3.1.1. Preparation and Pre-Swelling Behaviour of SHVE-M

**Figure 5:**
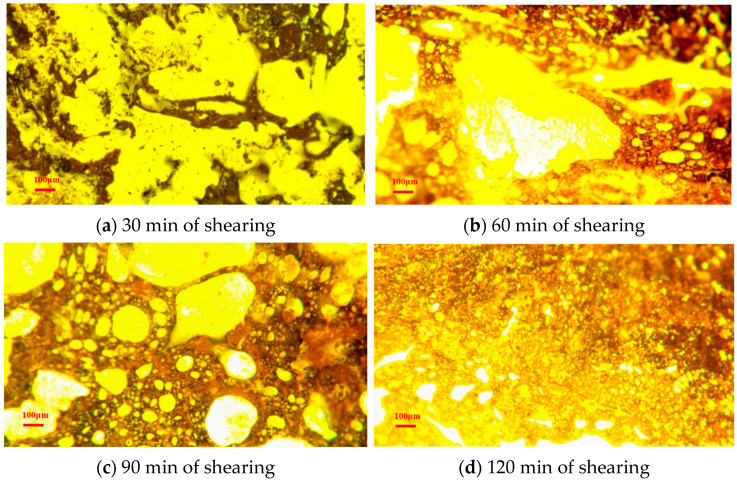

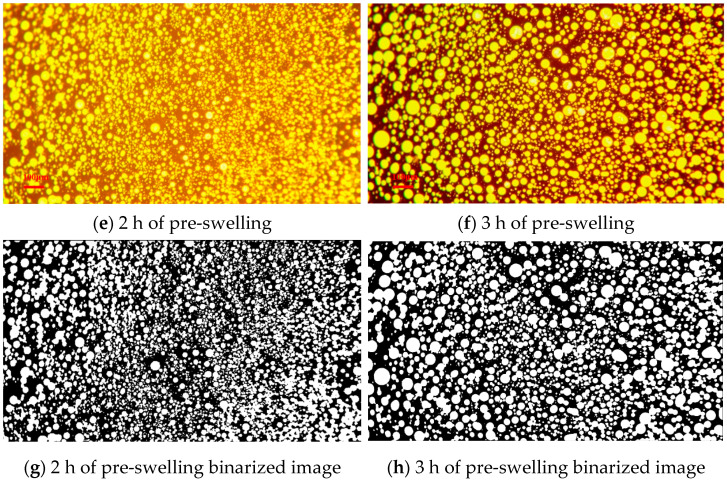
Fluorescence images of SHVE-M during preparation.

The preparation of SHVE-M is carried out sequentially through four stages: polymer dispersion, composite modification, swelling maturation, and extrusion granulation. The detailed procedure is as follows: First, 70# base bitumen is heated to 160 °C until fully molten and maintained at this temperature. Subsequently, accurately weighed SBS is added in 3–5 portions at 20 min intervals, followed by high-speed shearing at 3000 r/min for 90 min. Next, the predetermined amount of composite tackifier is introduced all at once, and mixing continues at the same speed for an additional 30 min to ensure uniform dispersion. The resulting mixture is then transferred to a constant-temperature oven set at 180 °C and allowed to undergo swelling and maturation for 3 h. Finally, the oven is gradually cooled to 80 °C; upon reaching thermal equilibrium, the material is fed into a single-screw extruder (screw speed: 120 r/min, barrel temperature: 80 °C–100 °C) for extrusion. The extrudate is subsequently water-cooled, shaped, and pelletized to yield uniform SHVE-M particles. FM enables the excitation of polymers such as SBS and composite tackifiers, inducing strong fluorescence emission that contrasts sharply with the dark bitumen phase, thereby facilitating high-resolution imaging [23,45]. FM observation of the microstructural evolution of SHVE-M at critical stages of preparation (Figure 5) clearly captures the dynamic changes associated with its pre-swelling behaviour.

Figure 5a presents the fluorescence image obtained after 30 min of shearing. At this stage, the SBS is initially fragmented, with large agglomerates remaining, leading to a non-uniform dispersion. The polymer phase (appearing as bright regions) manifests in irregular block-like structures with poorly defined interfaces relative to the bitumen phase (dark regions). Figure 5b illustrates the sample after 60 min of shearing. With extended shearing time, certain fragmented SBS microparticles absorb light components (saturates and aromatics) from the bitumen, causing them to swell and evolve into circular morphologies. These structures represent early-stage spherical precursors of the polymer phase. Nevertheless, the majority of microparticles retain irregular shapes. Figure 5c displays the microstructure following 90 min of shearing. Most of the SBS has been fragmented and swollen into spherical precursors; however, numerous incompletely dispersed agglomerates persist, indicating that further improvement is still required to achieve uniform distribution of the polymer phase. Figure 5d depicts the microstructural state after 120 min of shearing. During this stage, the composite tackifier effectively fills the interstitial spaces between the polymer and bitumen phases. Prolonged shearing further degrades the SBS, promoting its interpenetration with the tackifier and enabling synergistic dispersion, thereby achieving initial maturation. The bright polymer-rich phase (comprising SBS and tackifier) becomes predominant, exhibiting significantly enhanced dispersion and spatial distribution. Figure 5e illustrates the microstructure following 2 h of polymer swelling. Through isothermal incubation, the polymer components gradually absorb light fractions from the bitumen, initiating the formation of micron-scale spherical domains, representing the early-stage pre-swelling morphology. However, these spherical structures exhibit limited expansion, appear slightly contracted, and are characterised by poorly defined phase boundaries. Figure 5f presents the final microstructure after 3 h of pre-swelling, at which point SHVE-M has reached full maturation. The polymer spheres have evolved into more regular, voluminous, and morphologically well-developed structures. The phase contrast between bitumen and polymer is markedly improved, resulting in a uniformly distributed “bitumen phase–polymer spherical phase” composite system. This phase clearly proves the successful realisation of the desired pre-swelling effect, establishing a robust microstructural foundation for the subsequent rapid swelling process when combined with SBS-MB.

To further quantitatively characterize the microstructural evolution during the pre-swelling maturation stage, the fluorescence images at 2 h and 3 h were binarized (Figure 5g,h) and analyzed, with specific parameters listed in Table 9. As indicated in Table 9, after 2 h of pre-swelling, the modifier area fraction is 42.45%, accompanied by a particle count of 2736 and an average area fraction of 0.15‰. This quantitative result reflects the morphological observation that while the polymer has begun to swell, it retains relatively smaller individual dimensions. However, upon extending the maturation to 3 h, the modifier area fraction increases to 46.68%, suggesting the continuous absorption of light fractions by the polymer network. Notably, while the total modifier area increased, the number of microparticles decreased to 2044, and the average area fraction rose to 0.22‰. This inverse trend between particle count and average size implies a “coalescence and growth” process: as pre-swelling proceeds, smaller particles appear to expand and merge into larger spherical domains. These data indicate that the 3 h maturation period contributes to optimizing the pre-swelling effect, facilitating the formation of the “bitumen phase–polymer spherical phase” structure.

#### 3.1.2. Rapid Swelling Behaviour of SHVE-MB

**Figure 6:**
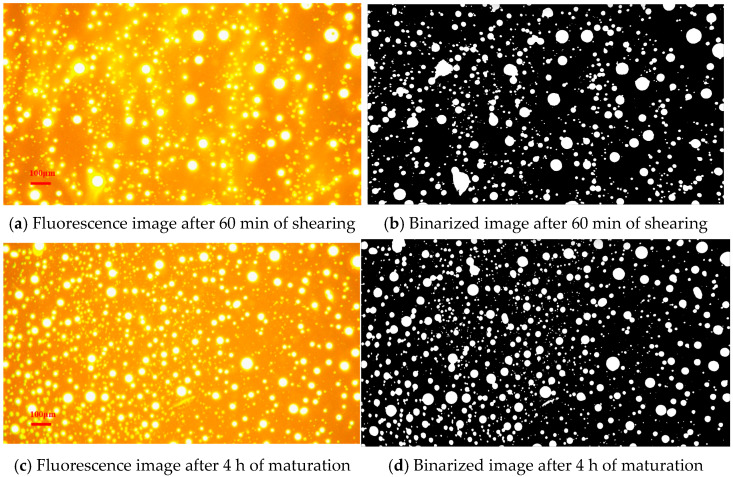
Fluorescence images of HVE-MB during preparation.

To investigate the mechanism that is responsible for the rapid swelling behaviour of SHVE-MB, Figure 6 displays fluorescence and binarized images captured at key stages of the HVE-MB preparation process (see Section 2.3). As illustrated in Figure 6a, after 60 min of shearing, the polymer phase exhibits a spherical or block-like morphology, with large, densely packed agglomerates presented in certain regions. A diffuse gradient transition zone is observed between the polymer and bitumen phases, indicating an ill-defined phase boundary. Additionally, some areas appear as “blank” zones devoid of polymer, revealing incomplete dispersion. This observation is further corroborated by the binarized image in Figure 6b: the white regions, representing the polymer phase, retain the spherical morphology but predominantly appear as partially coalesced “clusters” with irregular, serrated boundaries, along with numerous under-swelling domains. Fully swelling, well-dispersed spherical microparticles are scarcely observed. Quantitative analysis presented in Table 10 reveals a modifier area fraction of only 14.22%, comprising 1029 detected micro-particles, with an average area fraction of merely 0.13‰. These results indicate that 60 min of shearing achieves only mechanical fragmentation, resulting in a significant population of under-swelling microparticles and a microstructure characterised by non-uniform dispersion and insufficient swelling.

After two hours of isothermal maturation, significant microstructural transformations are observed in HVE-MB. As illustrated in Figure 6c, the polymer phase has completely separated from large agglomerates and evolved into uniformly sized, regularly shaped small spherical or quasi-spherical microparticles. Only minimal aggregation is evident among a few particles, and a distinct phase interface is formed between the polymer and bitumen phases, characterised by the absence of a blurred transition zone. The spherical microparticles are homogeneously dispersed throughout the bitumen, with no “blank areas” present. In the binarized image (Figure 6d), the white regions corresponding to the polymer phase appear as isolated, well-defined dots with smooth boundaries, randomly yet uniformly distributed against the black bituminous background. Notably, no interconnections between these domains are observed, indicating complete elimination of agglomeration. Consistent with the data presented in Table 10, the modifier area fraction increases to 17.79%, a rise of 3.57 percentage points compared to the state after 60 min of shearing. Concurrently, the number of microparticles reaches 1161 (an increase of 132), and the average area fraction attains 0.15‰. These observations indicate that during the maturation process, polymer microparticles continuously absorb light components from the bitumen, undergoing a synergistic evolution comprising “further fragmentation-full swelling-morphological regularisation”. The refinement of microparticles and the uniformity of their dispersion are substantially enhanced, while individual particles become swollen, dense, and structurally stable, ultimately forming a well-organised composite system consisting of a continuous “bitumen phase–polymer spherical phase”. It is evident that the conventional shear-maturation approach necessitates an extended protocol of “60 min shearing + 4 h maturation” to achieve both uniform dispersion and sufficient swelling in HVE-MB.

**Figure 7:**
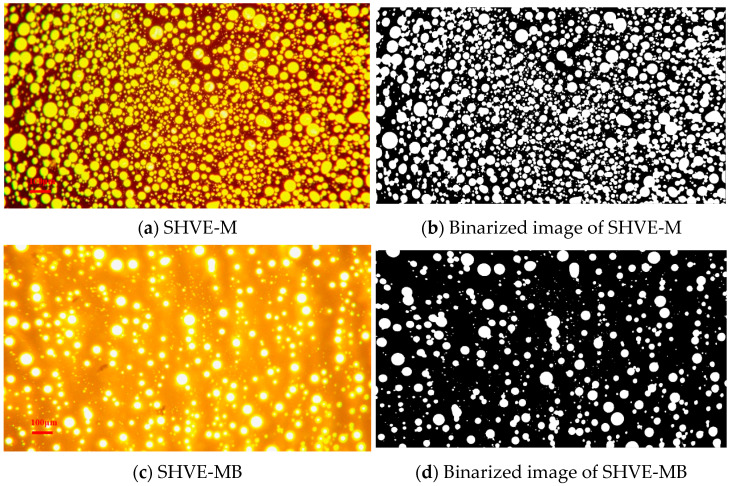
Fluorescence images of SHVE-MB during rapid swelling.

Figure 7a presents the fluorescence image of SHVE-M. Against the bitumen phase serving as the background, the polymer phase (comprising SBS and the composite tackifier) appears in the form of regularly spherical or quasi-spherical microparticles, uniformly dispersed throughout the bitumen phase. This morphology indicates complete and homogeneous swelling during sample preparation. The binarized image shown in Figure 7b reveals that the white regions (the polymer phase) are distinct, small dots with well-defined, smooth boundaries and no evidence of interconnected aggregates. These features collectively form a “uniformly dotted” pattern against the black bitumen phase. As further supported by the data in Table 10, the modifier area fraction of SHVE-M reaches 46.68%, with a total of 2044 microparticles detected and an average individual area fraction of 0.22‰. These results indicate a high number of well-dispersed, fully swelling polymer microparticles that are densely and stably embedded within the bitumen. This microstructure confirms excellent pre-swelling behaviour and provides a robust microstructural foundation for rapid swelling when applied as SBS-MB.

As shown in Figure 7c, after incorporating SHVE-M into SBS-MB and subjecting the mixture to 10 min of shearing, the resulting SHVE-MB exhibits microstructural characteristics highly similar to those of SHVE-M: the polymer phase remains predominantly spherical, with only a limited number of microparticles fractured into smaller, quasi-spherical domains due to shear forces. The distribution is uniform, indicating the formation of a stable composite with the bitumen phase. In the binarized image (Figure 7d), the white dots representing the polymer phase are slightly smaller than those observed in SHVE-M, yet their boundaries remain smooth and well-defined, without serrated or irregular fractures. According to Table 10, the modifier area fraction in SHVE-MB is 17.54%, significantly lower than the 46.68% observed in SHVE-M (as SHVE-M constitutes only 7% of SBS-MB), but closely comparable to that of HVE-MB (17.79%) after 4 h of conventional maturation. The average area fraction of individual polymer microparticles in SHVE-MB is 0.18‰, marginally higher than the 0.15‰ recorded for HVE-MB. This difference can be attributed to the fact that 10 min of shearing is sufficient to break only a small fraction of fully swelling polymer microparticles, while most of the pre-swelling polymer remains intact and effectively dispersed. The total microparticle count is 932 (lower than the 2044 observed in SHVE-M), yet still adequate to ensure a uniform dispersion throughout the bitumen phase. This microstructural analysis demonstrates that the pre-swelling polymer in SHVE-M can be efficiently dispersed within SHVE-MB through just 10 min of shearing, achieving a microstructural quality on par with that obtained via the conventional “60 min shearing + 4 h maturation” protocol. It is noteworthy that the pre-existing SBS network within the base SBS-MB plays a pivotal synergistic role in this rapid evolution. Unlike HVE-MB, which requires the construction of a polymer network from the ground up using base bitumen, the SHVE-MB system benefits from the initial SBS structure serving as a primary scaffold. The pre-swollen SHVE-M microparticles, acting as “reinforcing nodes”, can rapidly dock onto and bridge the existing polymer chains through the principle of compatibility. This “network grafting” mechanism significantly reduces the kinetic barrier for dispersion and swelling, allowing SHVE-MB to achieve a robust cross-linked structure comparable to HVE-MB within a fraction of the processing time.

**Figure 8:**
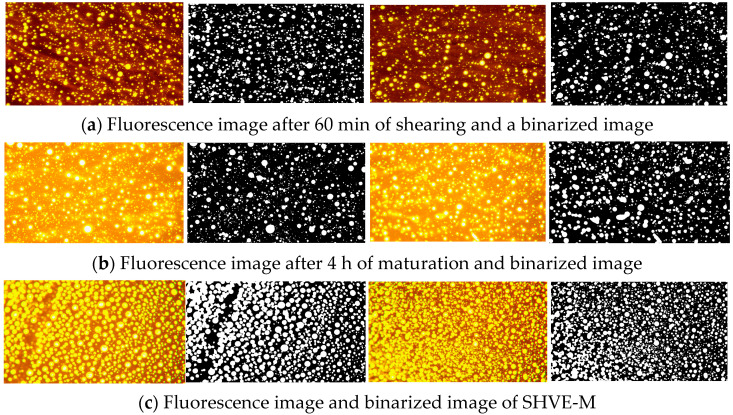

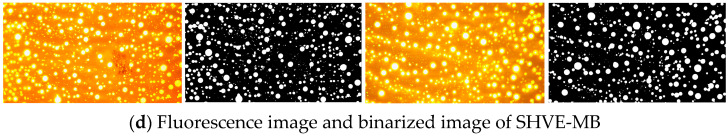
Parallel trials of SHVE-MB during rapid swelling.

To further verify the representativeness of the observed micro-morphologies and exclude experimental contingency, supplementary verification tests were conducted based on the aforementioned procedure. The fluorescence and binarized images of two additional parallel trials are presented in Figure 8, while the quantitative results of the binarized images from parallel trials are shown in Table 11. As evidenced by the data, the key microstructural parameters, including average area fraction and area fractions, exhibited high consistency across all replicates with negligible deviation. These findings indicate that the “bitumen phase–polymer spherical phase” structure and its rapid swelling behaviour described herein are robust, reproducible, systematic regularities rather than accidental phenomena.

#### 3.1.3. Molecular Weight and Its Distribution (GPC)

**Figure 9:**
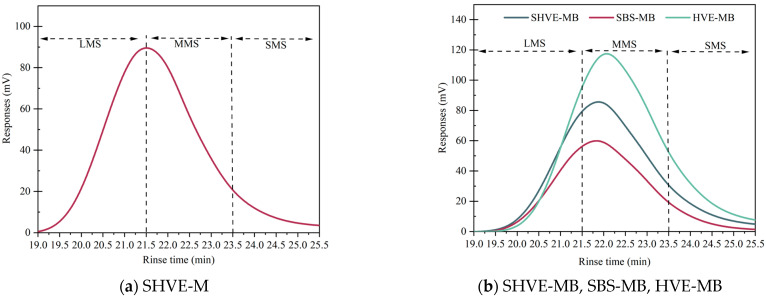
GPC test results.

GPC was employed to characterise the molecular size distribution and molecular weight of the bituminous system, providing critical molecular-level insights into the pre-swelling mechanism of SHVE-M. The results are presented in Figure 9 and Table 12. The elution time was divided into 13 equal intervals: fractions 1–5 correspond to large-sized molecules (LMS), 6–9 to medium-sized molecules (MMS), and 10–13 to small-sized molecules (SMS) [46]. It is important to acknowledge a key limitation of GPC analysis: while GPC effectively characterizes molecular size distribution and relative molecular weights, it does not directly measure physical crosslinking or network connectivity. The correlation between LMS content and polymer entanglement and network formation established in this study is based on indirect inference—chain entanglement during pre-swelling tends to increase the proportion of large-sized molecular aggregates, which is consistent with morphological observations (e.g., the formation of the “bitumen phase–polymer spherical phase” structure). Thus, LMS content is considered a reasonable proxy for effective swelling and mechanical reinforcement in this specific system, as SHVE-M’s pre-swelling primarily involves physical entanglement of SBS and composite tackifier chains rather than chemical crosslinking.

As shown in Figure 9 and Table 12, the LMS content directly reflects the extent of polymer chain entanglement and is closely associated with the pre-swelling treatment of SHVE-M. In Section 3.1.1, following “120 min shearing + 3 h pre-swelling maturation”, SBS and the composite tackifier form an interpenetrating network through chain interpenetration and entanglement, resulting in an LMS content of 43% in SHVE-M, thereby establishing the molecular foundation of the “bitumen phase–polymer spherical phase” morphology. In Section 3.1.2, after blending SHVE-M with SBS-MB under 10 min shearing, the LMS content of SHVE-MB reaches 29.2%, which is comparable to that of HVE-MB (30.7%). This indicates that the pre-swelling LMS aggregates in SHVE-M are only minimally disrupted during processing, enabling a similar level of chain entanglement to be achieved without undergoing the conventional “60 min shearing + 4 h maturation” procedure. Consequently, this approach avoids inefficient LMS formation caused by premature polymer agglomeration. Furthermore, this study intentionally adopted SBS-MB as the base binder for SHVE-MB. This choice is significant for the resulting molecular weight distribution. The pre-existing SBS network contributes a baseline LMS content, providing an already dispersed polymer environment. Consequently, SHVE-M circumvents the necessity for polymer components to undergo dispersion and reorganization to generate LMS structures, as is required for HVE-MB; instead, the pre-entangled LMS within SHVE-M co-disperses with the pre-existing LMS in the SBS-MB, collectively functioning within the SHVE-MB system. This mechanism explains why SHVE-MB can maintain a comparable LMS proportion (29.2%) to HVE-MB (30.7%) and achieve performance equivalence without the extensive maturation typically required to build molecular weight from scratch.

The polydispersity index (PDI) serves as an indicator of molecular chain size uniformity, with higher values corresponding to a more homogeneous molecular weight distribution. As presented in Table 12, SBS-MB exhibits a PDI of 1.898, which is lower than that of other samples, suggesting inferior dispersion uniformity. In contrast, SHVE-M achieves a well-defined spherical polymer phase and optimal dispersion uniformity through precise batch feeding, extended shearing, and constant-temperature maturation (see Section 3.1.1), resulting in a PDI of 2.065. This favourable microstructure is preserved during the rapid swelling process of SHVE-MB described in Section 3.1.2, as evidenced by a nearly unchanged PDI of 2.053. This indicates that the pre-swelling molecular architecture can withstand 10 min of shear treatment while maintaining excellent dispersion stability. Furthermore, the incorporation of SHVE-M significantly improves the dispersion uniformity of SBS-MB. Additionally, SHVE-MB demonstrates a slightly higher PDI compared to HVE-MB (1.950), reflecting enhanced dispersion of pre-swelling polymer chains within the bitumen. This observation is consistent with the findings summarised in Table 9 (see Section 3.1.2), where the polymer phase in SHVE-MB appears uniformly spherical and achieves a higher average area fraction (0.18‰) without 4 h of maturation, surpassing that of HVE-MB (0.15‰). In comparison, HVE-MB requires a more prolonged processing protocol, which is “60 min of shearing followed by 4 h of maturation”, to gradually break down agglomerates and attain optimized dispersion. The slightly lower PDI of HVE-MB is primarily attributed to the fact that, although chain entanglement (LMS) is adequately achieved in the conventional process, its efficiency in controlling uniformity is inferior to that of pre-swelling technology. This further underscores a key advantage of SHVE-M: the pre-organisation of molecular chains facilitates superior dispersion uniformity following brief shearing, thereby establishing a micro-molecular foundation for the enhanced macroscopic performance of SHVE-MB.

### 3.2. Properties of SHVE-MB and HVE-MB

#### 3.2.1. Physical Properties

**Figure 10:**
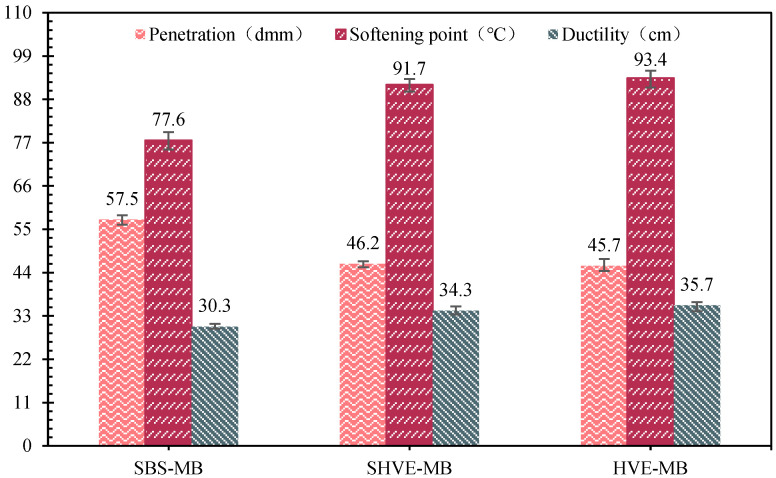
Physical properties of SHVE-MB and HVE-MB.

The physical properties of SHVE-MB, HVE-MB, and the reference SBS-MB are presented in Figure 10. Compared to SBS-MB, SHVE-MB exhibits a 19.7% lower penetration, a 18.2% higher softening point, and a 13.2% greater ductility. Its performance is highly comparable to that of HVE-MB, with only negligible differences of 0.5 dmm in penetration, 1.7 °C in softening point, and 1.4 cm in ductility. This indicates that the instant modification process achieves performance on par with the conventional method, without requiring extra shear treatment or maturation. This enhancement can be attributed to the pre-swelling effect of SHVE-M and the synergistic interaction of multiple modifiers. During the preparation of SHVE-M, the “shear dispersion-swelling maturation” stage is effectively completed in advance, resulting in uniformly dispersed and well-developed polymer spheres (comprising SBS and a composite tackifier) within the bitumen. This pre-formed “bitumen phase–polymer spherical phase” structure prevents polymer agglomeration and incomplete swelling that typically arises from short processing durations, thereby effectively overcoming the primary technical limitation associated with conventional bituminous modification. The composite tackifier in the SHVE-M system effectively fills the interfacial gaps between the polymer and bitumen, interpenetrates with SBS molecular chains to enhance cohesive strength, and restricts the flow of bitumen, thereby reducing penetration and increasing the softening point. The solubilizer, characterised by its high aromatic content, promotes the pre-swelling of SBS, facilitating the complete extension of PB soft segments and ensuring a uniform distribution of PS physical crosslinks, which significantly improves elastic recovery and ductility. Furthermore, the uniform dispersion of polymer spheres during the pre-swelling process prevents localised stress concentration, thereby enhancing the low-temperature ductility of SHVE-MB.

#### 3.2.2. Viscoelastic Properties

**Figure 11:**
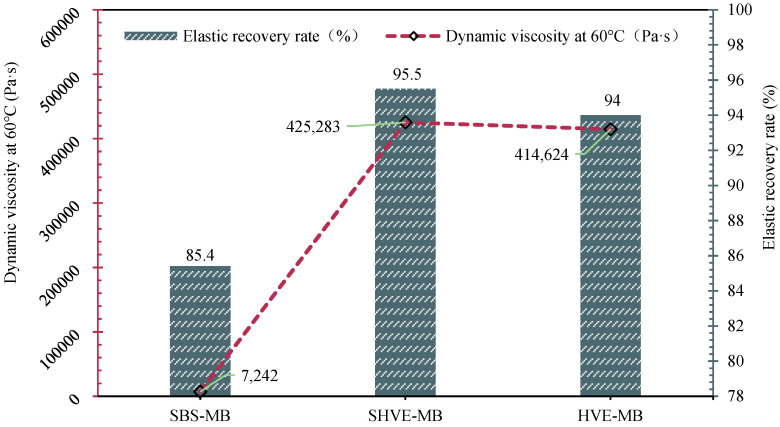
Viscoelastic properties of SHVE-MB and HVE-MB.

Viscoelastic tests reveal that (Figure 11), at 60 °C, the dynamic viscosities of SBS-MB, SHVE-MB, and HVE-MB are 7241.7 Pa·s, 425,283.4 Pa·s, and 414,623.7 Pa·s, respectively, with corresponding elastic recovery rates of 85.4%, 92.1%, and 93.6%. Compared to SBS-MB, SHVE-MB exhibits a 57.6-fold increase in dynamic viscosity and a 7.8% improvement in elastic recovery. Its viscosity is slightly higher than that of HVE-MB by 10,659.7 Pa·s, while its elastic recovery is only 1.5 percentage points lower. These results indicate that the instant-modified bitumen achieves performance levels comparable to, or even exceeding, those of conventional high-viscosity, high-elasticity bitumen in terms of bonding strength and elastic recovery. This enhancement is attributed to the pre-swelling of SHVE-M and the synergistic effects of multiple modifiers. The pre-swelling mechanism facilitates the formation of well-developed spherical polymer structures within SHVE-M, which rapidly disperse into SBS-MB without requiring additional maturation time, thereby establishing a continuous three-dimensional elastic network. This physically crosslinked architecture effectively restricts the flow of bituminous binder, significantly improving resistance to high-temperature deformation and consequently enhancing the dynamic viscosity at 60 °C. From the perspective of modifier effects, the PB soft segments of SBS fully absorb the saturates and aromatics presented in bitumen during the pre-swelling stage. Upon expansion, these segments form an interpenetrating network with the composite tackifier, thereby enhancing the system’s elastic recovery. Waste PE powder acts as “rigid support points” within this network, further improving bonding strength [47]. Moreover, the solubilizer enhances the compatibility between SBS and bitumen, effectively preventing polymer agglomeration and ensuring stable dynamic viscosity and superior elastic recovery, both properties outperforming those of SBS-MB [48]. In contrast to conventional HVE-MB, which requires 60 min of shearing followed by a 4 h maturation period to develop its network structure, SHVE-M benefits from a pre-swelling effect that pre-forms a stable dispersion, resulting in negligible viscoelastic differences.

#### 3.2.3. Rheological Properties

**Figure 12:**
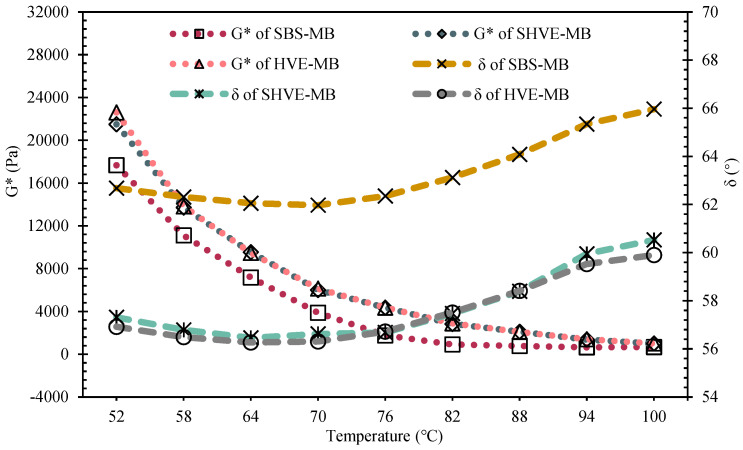
Complex shear modulus (G*) and phase angle (δ) of SHVE-MB and HVE-MB.

**Figure 13:**
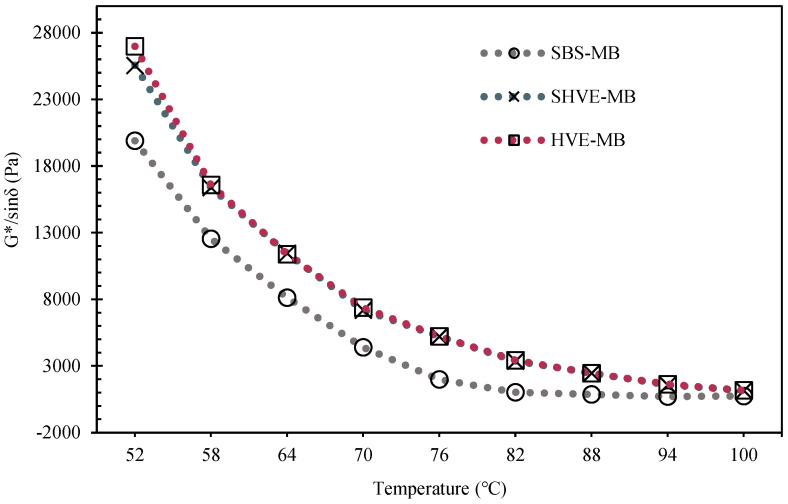
Rutting factor (G*/sinδ) of SHVE-MB and HVE-MB.

As shown in Figure 12, within the temperature range of 52–100 °C, the phase angles (δ) follow the trend: SBS-MB > SHVE-MB > HVE-MB. The phase angle of SHVE-MB is on average 5.8° lower than that of SBS-MB and differs from HVE-MB by no more than 0.8°. In contrast, the rutting factor (G*/sinδ) follows the order: HVE-MB > SHVE-MB > SBS-MB (Figure 13), with SHVE-MB exhibiting a 142.3% increase compared to SBS-MB and differing from HVE-MB by only 3.2%. According to the fitting equations listed in Table 13, the failure temperatures of SBS-MB, SHVE-MB, and HVE-MB are determined to be 82 °C, 101.8 °C, and 101.6 °C, respectively. These results indicate that SHVE-MB possesses a significantly higher proportion of elastic components and superior high-temperature rutting resistance relative to SBS-MB, performing nearly as well as HVE-MB. The enhanced rheological properties can be attributed to the pre-swelling mechanism of SHVE-M and the synergistic effects of the modifiers. During the preparation of SHVE-M, following “120 min shearing + 3 h maturation”, the polymer phase (comprising SBS and a composite tackifier) develops into fully swollen, regularly spherical structures. This pre-formed microstructure enables SHVE-M to rapidly establish a uniform “bitumen phase–polymer spherical phase” system within SBS-MB after only 10 min of shearing, thereby avoiding the polymer agglomeration typically observed in the early stages of conventional HVE-MB production. Within the tested temperature range, the PS hard segments of SBS impart consistent rigidity, which, combined with physical crosslinking induced by the composite tackifier, establishes a “rigid support-elastic buffer” architecture. This structural feature enhances the resistance of SHVE-MB to repeated shear deformation, as evidenced by an increase in the complex shear modulus (G*). Pre-swelling ensures uniform dispersion of the polymer, minimizes network defects, and improves the elastic response, reflected in a reduced phase angle (δ), thereby enhancing the rutting factor (G*/sinδ). The solubilizer further reinforces interfacial adhesion by promoting the swelling and fusion of SBS with bitumen, effectively preventing high-temperature rheological deterioration caused by polymer phase separation. Compared to conventional SBS-MB, SHVE-MB exhibits more stable rheological behaviour and superior resistance to high-temperature deformation [49]. The minor performance difference between SHVE-MB and HVE-MB primarily stems from more complete polymer swelling in the latter due to extended maturation time. However, the pre-swelling technology employed in SHVE-MB significantly narrows this gap, enabling the integration of “short-time preparation–high performance”.

### 3.3. Pavement Performance

#### 3.3.1. High-Temperature Stability

**Figure 14:**
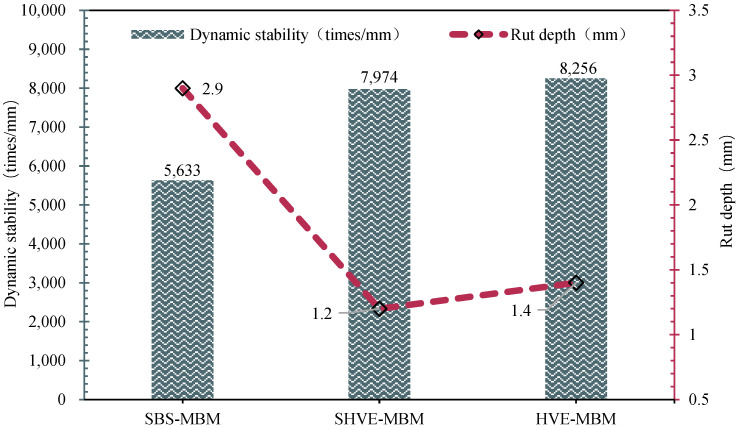
Rutting test results.

The rutting test results, presented in Figure 14, illustrate the high-temperature deformation resistance of the three bituminous mixtures. The SBS-MBM exhibits a low DS value of 1358 times/mm and a pronounced rut depth, whereas the SHVE-MBM achieves a significantly higher DS value of 7974 times/mm, accompanied by markedly reduced rutting. In comparison with the conventionally prepared HVE-MBM, which has a DS value of 8256 times/mm, the SHVE-MBM shows only a minor difference of 282 times/mm, with nearly equivalent rut depths. These findings indicate that the instantly modified bitumen substantially enhances high-temperature rutting resistance, outperforming the SBS-MBM and approaching the performance level of HVE-MBM.

This conclusion arises from the macroscopic manifestation of SHVE-M’s pre-swelling mechanism and the synergistic effects of its modifiers. On the one hand, during the preparation of SHVE-M, the “shear dispersion–swelling maturation” process is already completed, resulting in well-developed and fully swelling polymer spherical phases composed of SBS and a composite tackifier. When blended with SBS-MB and subjected to only 10 min of shearing, these pre-swelling phases rapidly establish a continuous and uniform three-dimensional elastic network within the bitumen, without requiring additional maturation. This network functions as a “rigid framework”, providing physical crosslinking that significantly restricts colloidal flow of the bitumen at elevated temperatures and resists plastic deformation under repeated loading, thereby markedly enhancing the dynamic stability [50]. On the other hand, the crystalline structure of waste PE powder within the composite tackifier introduces “rigid support points” that act in synergy with the PB soft segments of SBS, further improving resistance to high-temperature deformation [51]. Additionally, the solubilizer enhances compatibility between the polymer and bitumen, preventing localised agglomeration and thus mitigating the uneven rutting issues. In contrast to conventional HVE-MBM, which necessitates a prolonged processing protocol of “60 min shearing + 4 h maturation” to achieve a stable network, SHVE-M’s pre-swelling technology enables complete polymer dispersion from the outset, attaining equivalent high-temperature performance with merely 10 min of shearing.

#### 3.3.2. Water Stability

Water stability tests assess resistance to water damage through two key indicators: the MSR and the TSR. The results are presented in Table 14. As shown in Table 14, SBS-MBM exhibits an MSR of 84.5% and a TSR of 85.6%, indicating limited resistance to water damage and a propensity for interfacial delamination and strength degradation under wet or freeze–thaw conditions. In contrast, SHVE-MBM achieves an MSR of 96.3% and a TSR of 97.4%. When compared to HVE-MBM, which has an MSR of 98.8% and a TSR of 98.0%, the differences are only 2.5 and 0.6 percentage points, respectively, demonstrating that the water damage resistance of SHVE-MBM is nearly equivalent to that of HVE-MBM.

This outstanding performance stems from the dual optimisation effect of pre-swelling technology on the interfacial structure and compactness of bituminous mixtures. At the microscale, the pre-swelling spherical polymer phases in SHVE-M are uniformly dispersed within the bitumen phase. During mixing, these microparticles generate a “three-dimensional encapsulation effect” on aggregate surfaces, promoting more uniform and robust bituminous adhesion. This markedly increases the effective contact area, strengthens interfacial bonding, and effectively mitigates water-induced interfacial debonding. Concurrently, the waste PE powder incorporated in the composite tackifier exhibits high hydrophobicity. When homogeneously distributed throughout the bituminous film, it reduces surface hydrophilicity and restricts both water adsorption and penetration at the interface. The continuous elastic network formed through pre-swelling enhances the film’s toughness and self-healing capability, enabling resistance to microcrack propagation caused by volumetric changes and preventing film delamination under conditions of water immersion or freeze–thaw cycling [52]. Furthermore, pre-swelling technology effectively mitigates polymer agglomeration by proactively eliminating internal voids and minimising water penetration pathways in SHVE-MBM, thereby yielding a denser microstructure and substantially improving resistance to water erosion. In contrast, conventional HVE-MBM relies on extended shearing and maturation periods to achieve adequate polymer swelling and uniform dispersion, which are essential for achieving satisfactory water stability. SHVE-M, however, incorporates pre-swelling to expedite both polymer swelling and dispersion, facilitating the rapid development of a compact and stable “bitumen–polymer-aggregate” interfacial network. As a result, the mixture achieves water stability comparable to that attained through the conventional process.

#### 3.3.3. Low-Temperature Crack Resistance

The low-temperature SCB test is employed to assess the crack resistance of bituminous mixtures through two key indicators: the $\sigma_{max}$ and the $K_{IC}$. As presented in Table 15, SBS-MBM exhibits a maximum load of 9300 N, a fracture strength of 1.4 MPa, and a fracture toughness of 27.9 N/mm^1.5^. In contrast, SHVE-MBM achieves significantly higher values of 13,520 N, 2.0 MPa, and 39.8 N/mm^1.5^, representing improvements exceeding 42%. The differences between SHVE-MBM and HVE-MBM, 14,500 N, 2.2 MPa and 43.9 N/mm^1.5^, are merely 980 N, 0.2 MPa, and 4.1 N/mm^1.5^, respectively, indicating nearly equivalent low-temperature performance.

The superior low-temperature performance of SHVE-MBM is quantitatively reflected in the SCB test results. While $\sigma_{max}$ indicates the peak load-bearing capacity, $K_{IC}$ offers a more critical insight into the material’s intrinsic ability to resist crack propagation under brittle conditions. As shown in Table 15, SHVE-MBM exhibits a $K_{IC}$ of 39.8 N/mm^1.5^, representing a substantial 42.6% enhancement over SBS-MBM. This suggests that the energy threshold required for crack advancement has been significantly elevated. This enhancement is likely associated with the stable “bitumen phase–polymer spherical phase” microstructure formed by pre-swelling technology, which rapidly develops into a continuous three-dimensional elastic network after only 10 min of shearing.

Mechanistically, this microstructure appears to activate three key toughening mechanisms to inhibit failure. First, crack deflection: The uniformly dispersed, pre-swollen polymer spheres act as rigid yet elastic obstacles. Instead of cutting directly through the polymer phase, the crack front tends to tilt and twist around these high-modulus domains. This deviation forces the crack to propagate along a longer, non-planar path (increasing tortuosity). Second, crack bridging: The pre-swelling process facilitates the entanglement of polymer chains, indicated by the high proportion of large molecular size (LMS) fraction. As the crack opens, these entangled chains span the crack surfaces, functioning as “micro-bridges”. These bridges exert a pulling force that opposes the crack opening, thereby helping to shield the crack tip from the full magnitude of the applied stress. The composite tackifier further interpenetrates the SBS chains to enhance this bridging capacity, while the solubilizer improves phase compatibility to mitigate interfacial debonding. Third, energy dissipation: PB soft segments within the spherical phases undergo significant viscoelastic deformation in the process zone ahead of the crack tip. Through molecular relaxation—the process where polymer chains reorient and untangle to relieve stress—the stored elastic strain energy is converted into heat. This dissipation contributes to the blunting of the sharp crack tip, thus inhibiting further crack growth. In contrast to the poorly dispersed agglomerates in SBS-MBM, which may serve as stress concentrators, the homogeneous network in SHVE-MBM effectively integrates these mechanisms, resulting in low-temperature crack resistance comparable to the mature network found in HVE-MBM.

### 3.4. Limitations and Future Outlook

Although this study successfully validates the pre-swelling mechanism of SHVE-M and verifies the superior performance of the resulting SHVE-MB and SHVE-MBM through a “micro-mechanism with macro-performance” framework, there are still limitations that warrant further investigation to fully bridge the gap between laboratory characterisation and long-term engineering applications.

Firstly, regarding the mechanical fracture behaviour observed in the SCB tests, this study primarily relied on experimental data to explain the toughening mechanisms (crack deflection, bridging, and energy dissipation). While effective, this phenomenological explanation lacks a quantitative numerical verification. Recent advances in computational mechanics suggest that numerical modelling strategies, such as the Finite Element Method (FEM) incorporating Cohesive Zone Models (CZMs) or damage-based approaches, can provide profound insights into damage evolution and crack propagation in complex composites [53,54,55]. Future research should focus on establishing a multi-scale numerical model capable of reproducing the interaction between the “bitumen phase–polymer spherical phase” microstructure and the aggregate skeleton. Simulating the stress concentration and dissipation at the interface of pre-swollen polymer spheres would theoretically validate the enhanced $K_{IC}$ observed in this work.

Secondly, for the rheological properties of high-performance modified bitumen, the G*/sinδ derived from DSR tests was employed in this study to ensure consistency with current standard specifications and to provide a direct comparison with the control groups (SBS-MB and HVE-MB). However, it is acknowledged that G*/sinδ, measured within the linear viscoelastic (LVE) region, may not fully capture the high-temperature deformation resistance of highly polymer-modified binders under heavy traffic loads and large strains. The Multiple Stress Creep Recovery (MSCR) test is increasingly recognised as a more effective method to evaluate the non-linear viscoelastic response and rutting resistance of modified bitumen. In future studies, MSCR testing will be conducted to obtain non-recoverable creep compliance and percent recovery parameters, providing a more comprehensive assessment of SHVE-MB’s performance under extreme loading conditions.

Finally, regarding the molecular weight distribution, this study adopted the widely accepted GPC fraction classification (LMS/MMS/SMS) [56,57]. However, since SHVE-M is a multi-component composite system, the precise applicability of this standard classification to such complex modifiers remains to be further verified. Future studies will focus on validating the correlation between these molecular fractions and the chemical composition of composite-modified bitumen.

## 4. Conclusions

This study systematically investigated the pre-swelling mechanism of SHVE-M through microscopic analyses, elucidating its behaviour in bitumen. By integrating macroscopic evaluations of physical properties, rheological performance, and pavement performance, a coupled verification framework was established, namely “micro-mechanism with macro-performance”. The main conclusions are shown as follows:

(1) FM observations revealed that after “120 min shearing + 3 h pre-swelling maturation”, SHVE-M forms a composite system characterised by a “bitumen phase–polymer spherical phase” morphology, with the polymer phase presenting as regular and plump spherical structures uniformly dispersed. GPC results indicated that its LMS accounts for 43% with a PDI of 2.065, indicating uniform molecular chain entanglement. The modification process is dominated by physical interactions, with SHVE-M exhibiting good compatibility with bitumen and each component synergistically forming a stable microstructure, laying a solid foundation for rapid swelling and dispersion.

(2) Characterization via FM and Image J-based image analysis demonstrated that the preparation process of SHVE-M is simple and does not rely on complex production equipment, achieving uniform dispersion with only 10 min of shearing. Its modifier area fraction is 17.54% and average area fraction is 0.18‰, which is essentially consistent with the microscopic distribution of HVE-MB after “60 min shearing + 4 h maturation”.

(3) SHVE-MB was prepared by adding 7% SHVE-M to SBS-MB, requiring no additional swelling or maturation stage and being formable via only 10 min of mechanical shearing. Its performance is as follows: penetration of 46.2 dmm, softening point of 91.7 °C, and ductility of 34.3 cm, which are highly comparable to those of HVE-MB prepared via the conventional process; dynamic viscosity at 60 °C reaches 425,283.4 Pa·s with an elastic recovery rate of 92.1%, showing viscoelastic properties superior to conventional high-viscosity and high-elasticity bitumen; rheological properties are significantly optimized that the δ is reduced by an average of 5.8° compared to SBS-MB, the G*/sinδ is increased by an average of 142.3%, the failure temperature is 101.8 °C, and high-temperature deformation resistance is substantially enhanced.

(4) Compared to SBS-MBM, the pavement performance of SHVE-MBM is significantly improved and comparable to that of conventional HVE-MBM: high-temperature DS reaches 7974 times/mm with a markedly reduced rut depth; it exhibits excellent water stability, with a MSR of 96.3% and a TSR of 97.4%, demonstrating outstanding resistance to water damage; in the low-temperature SCB test at −10 °C, its maximum load is 13,520 N, $\sigma_{max}$ is 2.0 MPa, and K_IC_ is 39.8 N/mm^1.5^, representing an increase of over 42% in low-temperature crack resistance compared to SBS-MBM.

(5) SHVE-MB meets or even slightly exceeds the performance of conventional wet-process modified bitumen, particularly in terms of viscoelastic properties. It effectively bridges the performance gap between instant modified bitumen and conventional wet-process modified bitumen, vigorously promoting the technological development of instant modifiers, achieving true rapid swelling and high-performance reconstruction, and providing a solid theoretical basis and feasible technical solutions.

## Figures and Tables

**Table 1:** Technical properties of base bitumen.

Items	Unit	Test Result	Testing Regulation
Penetration (25 °C, 100 g, 5 s)	dmm	78.4	ASTM D5 [35]
Softening point (Ring and Ball)	°C	46.1	ASTM D36 [36]
Ductility (15 °C, 50 mm/min)	cm	≥100	ASTM D113 [37]
Dynamic viscosity (60 °C)	Pa·s	203	ASTM D4402 [38]

**Table 2:** Technical properties of solubilizer.

Items	Unit	Test Result	Testing Regulation
Density (20 °C)	g/cm^3^	1.08	ASTM D1505 [39]
Flash point (20 °C)	-	200	ASTM D93 [40]
Viscosity (100 °C)	mm^2^/s	16	ASTM D445 [41]

**Table 3:** Technical properties of SBS.

Items	Unit	Test Result
Elongation at break	%	717
Volatile content (mass fraction)	%	0.07
Tensile strength	MPa	16.4

**Table 4:** Technical properties of waste PE powder.

Items	Unit	Test Result	Testing Regulation
Density (15 °C)	g/cm^3^	0.96	ASTM D1505
Melt flow rate	g/10 min	4.4	ASTM D1238 [42]
Yield strength	MPa	10	ISO 527 [43]
Breaking strength	MPa	15	ISO 527
Elongation at break	%	770	ISO 527

**Table 5:** Technical properties of SBS-MB.

Items	Unit	Test Result	Testing Regulation
Penetration (25 °C, 100 g, 5 s)	dmm	57.5	ASTM D5
Softening point (Ring and Ball)	°C	77.6	ASTM D36
Ductility (15 °C, 50 mm/min)	cm	30.3	ASTM D113
Dynamic viscosity (60 °C)	Pa·s	7241.7	ASTM D4402
Elastic recovery rate (25 °C)	%	85.4	JTG 3410 T0662 [44]

**Table 6:** Technical properties of coarse aggregate.

Items		Unit	5–10 mm	3–5 mm
Apparent relative density		-	2.950	2.866
Water absorption		%	0.7	0.8
Content of particles less than 0.075 mm		%	0.3	0.2
Content of needle-like and flaky particles	Particle size > 9.5 mm	%	-	-
	Particle size < 9.5 mm	%	6.2	-

**Table 7:** Technical properties of fine aggregate.

Items	Unit	0–3 mm
Apparent relative density	-	2.695
Soundness (fraction > 0.3 mm)	%	4.7
Sand equivalent	%	74
Methylene blue value	g/kg	0.9
Angularity (flow time)	s	45.6

**Table 8:** Technical properties of mineral powder.

Items	Unit	Test Result
Apparent density	g/cm^3^	2.730
Moisture content	%	0.2
Hydrophilic coefficient	-	0.4
Plasticity index	%	2.8
Thermal stability	-	No obvious change

**Table 9:** Quantitative results of binarized images under different pre-swelling times.

Sample	Area Fraction	Number of Microparticles	Average Area Fraction
3 h of pre-swelling binarized image	46.68%	2044	0.22‰
2 h of pre-swelling binarized image	42.45%	2736	0.15‰

**Table 10:** Quantitative results of binarized images.

Sample	Area Fraction	Number of Microparticles	Average Area Fraction
SHVE-M	46.68%	2044	0.22‰
SHVE-MB	17.54%	932	0.18‰
HVE-MB (60 min of shearing)	14.22%	1029	0.13‰
HVE-MB (4 h of maturation)	17.79%	1161	0.15‰

**Table 11:** Quantitative results of parallel trials.

Sample	Area Fraction	Number of Microparticles	Average Area Fraction
HVE-MB (60 min of shearing)	13.63%	1090	0.12‰
	13.46%	1016	0.13‰
HVE-MB (4 h of maturation)	17.98%	1201	0.15‰
	18.64%	1331	0.14‰
SHVE-M	45.66%	2029	0.22‰
	45.21%	2132	0.21‰
SHVE-MB	16.96%	893	0.18‰
	16.85%	851	0.19‰

**Table 12:** Quantitative results of GPC tests.

Simple	LMS (%)	MMS (%)	SMS (%)	Mw (g/mol)	Mn (g/mol)	PDI
SHVE-M	43	49.8	7.2	6547	3171	2.065
SBS-MB	25.3	56.4	18.3	4090	2154	1.898
SHVE-MB	29.2	58.7	12.1	4941	2407	2.053
HVE-MB	30.7	53.6	15.7	5309	2722	1.950

**Table 13:** Linear fitting results of G*/sinδ.

Sample	Fitting Equation	R^2^
SHVE-MB	y = 746,034.42 × e^−0.065x^	0.998
HVE-MB	y = 821,361.15 × e^−0.066x^	0.998

**Table 14:** Water stability test results.

Simple	MSR (%)	TSR (%)
SBS-MBM	84.5	85.6
SHVE-MBM	96.3	97.4
HVE-MBM	98.8	98.0

**Table 15:** SCB test results at −10 °C.

Simple	$F_{max}$ (N)	$\sigma_{max}$ (MPa)	$K_{IC}$ (N/mm^1.5^)
SBS-MBM	9300	1.4	27.9
SHVE-MBM	13,520	2.0	39.8
HVE-MBM	14,500	2.2	43.9

## Data Availability

The original contributions presented in this study are included in the article. Further inquiries can be directed to the corresponding author.
